# Eculizumab Treatment of Massive Hemolysis Occurring in a Rare Co-Existence of Paroxysmal Nocturnal Hemoglobinuria and Myasthenia Gravis

**DOI:** 10.3390/hematolrep16020025

**Published:** 2024-04-19

**Authors:** Ráhel Réka Bicskó, Árpád Illés, Zsuzsanna Hevessy, Gergely Ivády, György Kerekes, Gábor Méhes, Tünde Csépány, Lajos Gergely

**Affiliations:** 1 Department of Hematology, Institute of Internal Medicine, Faculty of Medicine, Clinical Center, University of Debrecen, 4032 Debrecen, Hungary; illes.arpad@med.unideb.hu (Á.I.); lgergely@med.unideb.hu (L.G.); 2 Department of Laboratory Medicine, Faculty of Medicine, Clinical Center, University of Debrecen, 4032 Debrecen, Hungary; hevessy@med.unideb.hu (Z.H.); ivady@med.unideb.hu (G.I.); 3 Intensive Care Unit, Institute of Internal Medicine, Faculty of Medicine, Clinical Center, University of Debrecen, 4032 Debrecen, Hungary; gkerekes@med.unideb.hu; 4 Department of Pathology, Faculty of Medicine, Clinical Center, University of Debrecen, 4032 Debrecen, Hungary; gabor.mehes@med.unideb.hu; 5 Department of Neurology, Faculty of Medicine, Clinical Center, University of Debrecen, 4032 Debrecen, Hungary; csepany@med.unideb.hu

**Keywords:** paroxysmal nocturnal hemoglobinuria, myasthenia gravis, eculizumab, hemolysis

## Abstract

The co-occurrence of myasthenia gravis (MG) and paroxysmal nocturnal hemoglobinuria (PNH) is rare; only one case has been published so far. We report a 63-year-old Caucasian female patient who was diagnosed with MG at the age of 43. Thymoma was also detected, and so it was surgically resected, which resulted in reasonable disease control for nearly 20 years. Slight hemolysis began to emerge, and then myasthenia symptoms progressed, so immunosuppressive therapy was started. Due to progressive disease and respiratory failure, the patient underwent plasmapheresis, and ventilatory support was stopped. Marked hemolysis was present, and diagnostic tests confirmed PNH with type III PNH cells. Her myasthenia symptoms aggravated, mechanical ventilation had to be started again, and due to the respiratory acidosis, massive hemolysis occurred. After two plasmapheresis sessions, the patient received eculizumab at 600 mg, resulting in prompt hemolysis control. After the second dose of the treatment, the patient was extubated. Still, due to their inability to cough, she developed another respiratory failure and pneumonia–sepsis, resulting in the patient’s death. This case highlights the rare association between these two serious diseases and similar immune-mediated pathophysiology mechanisms involving the complement system.

## 1. Background

Myasthenia gravis is an autoimmune neuroinflammatory disorder where the immune reaction disrupts neuromuscular signal delivery due to autoantibodies against the acetylcholine receptors (AcRs). The damaging immune reaction heavily relies on the complement cascade [1]. Myasthenia is often the result of a persistent thymus or thymoma in the patient. The targeted inhibition of the complement cascade resulted in very promising results [2,3]. For the last 5 years, the use of eculizumab in refractory acetylcholine-receptor-positive myasthenia gravis has been an available treatment option based on the results of the REGAIN study [4,5]. Based on the promising results with eculizumab, additional complement-blocking therapies, like ravulizumab and zilucoplan, which is a macrocyclic C5 inhibiting peptide, are being investigated nowadays [6]. The treatment of myasthenia gravis may be challenging to the physician as acute crises may require prompt intervention, and patients frequently require intensive care support with mechanical ventilation due to respiratory muscle weakness. 

Paroxysmal nocturnal hemoglobinuria (PNH) is a disorder of the stem cells, where a somatic mutation in the X chromosome-located PIGA gene results in the lack or reduced expression of the anchor glycolipid GPI keeping CD55 and CD59 on the surface of blood cells. The decreased expression of these protective factors on erythrocytes makes them prone to spontaneous complement attack and hemolysis, leading to the typical clinical symptoms of the disease [7]. As a stem cell disorder, the association of PNH with aplastic anemia is also well characterized [8]. Sometimes, massive hemolysis occurs in the disease course, requiring immediate action. The treatment of PNH is mainly based on continuous complement-blocking therapy, mostly with eculizumab or ravulizumab [9]. In severe, refractory cases, an allogenic bone marrow transplantation can cure the disease [10].

The co-existence of myasthenia gravis and paroxysmal nocturnal hemoglobinuria is rare. Three cases have been published so far [11,12,13]. The common pathophysiology mechanism involving the complement system provides a unique opportunity to target both diseases with one drug. Here, we report a case of severe myasthenia with the co-occurrence of type III PNH cells, resulting in massive hemolysis.

## 2. Case Presentation

We present the case of a 63-year-old woman who suffered from several different immune-mediated diseases at the same time. She had been diagnosed with myasthenia gravis associated with thymoma in 2001. She was treated by thymectomy, and a histological examination confirmed type B1 thymoma, corresponding to the predominantly cortical type of Marino and a Muller-Hermelink classification. Antibodies to the acetylcholine receptor (anti-AChR) were detectable. Cholinesterase inhibitor therapy was initiated, and as a result, the disease was in remission for 6 years. In 2007, however, she experienced muscle weakness and difficulty in swallowing, and the cholinesterase inhibitor was supplemented with methylprednisolone therapy. Symptoms improved rapidly, so the steroid dose was tapered and then discontinued. She was in good health between 2007 and 2020, with 1–2 mild flares per year. Occasional flares were intermittently treated with corticosteroids and azathioprine. In 2019, an immunological examination was performed due to joint pain and Raynaud’s symptoms. Her laboratory tests demonstrated an elevated erythrocyte sedimentation rate and positive tests for antinuclear and anti-double-stranded DNA antibodies. This exhausted the criteria of undifferentiated autoimmune diseases. Chloroquine therapy was initiated, but after 1 year, the drug had to be stopped due to ophthalmic side effects. All the blood counts were unremarkable until 2021. In February 2021, mild hemolysis occurred with macrocytic anemia, an elevated lactate dehydrogenase level, an elevated reticulocyte count, and an undetectable serum haptoglobin level. She was still in good general condition. Thus, the exploration of the cause of hemolysis began in an outpatient setting. The hemoglobin level was not lower than 10 g/dL. However, from September 2021, she experienced the progression of muscle weakness with predominant bulbar symptoms. In October 2021, she was admitted to the hospital because of a flare with muscle weakness and respiratory failure. Oral corticosteroid therapy was initiated in combination with a high-dose cholinesterase inhibitor, but symptoms did not improve, and respiratory failure persisted; therefore, mechanical ventilation and plasmapheresis were required. Intermittent plasmapheresis was performed with complete plasma substitution. After 8 days of intensive care unit treatment, she was successfully extubated, and the pseudobulbar symptoms improved significantly. A hemolytic crisis developed during this flare, leading to transfusion-dependent anemia (hemoglobin at 7.1 g/dL, elevated lactate dehydrogenase—three-fold the normal value—and reticulocytosis 116 × 10^9^/L). Peripheral blood flow cytometry confirmed the diagnosis of PNH according to current guidelines [14]. The size of the PNH clone was 46% of erythrocytes and 45% of granulocytes; PNH with type III PNH cells was diagnosed.

**Figure 1:**
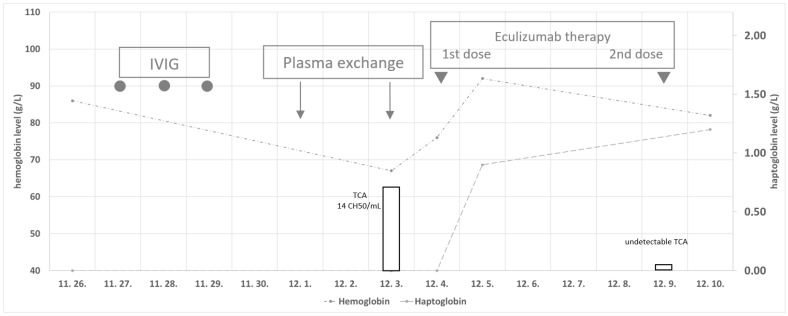
Changes in hemoglobin (g/L) and haptoglobin (g/L) levels over time and total complement activity with eculizumab treatment. IVIG—intravenous immunoglobulin. TCA—total complement activity.

A bone marrow trephine biopsy revealed slightly decreased cellularity with reduced hematopoietic activity and reactive cells. Myelodysplastic syndrome and malignancy could be ruled out. Low-molecular-weight heparin was started at a therapeutic dose to prevent thrombotic events. An application for an Individual Funding Request for eculizumab treatment was filed. During the observation, muscle weakness worsened again; therefore, the corticosteroid dose was increased, and IVIg treatment was started, with a total amount of 1 g/kg of body weight. However, her condition rapidly deteriorated, bulbar symptoms progressed again, global respiratory failure developed due to respiratory muscle weakness, and she required mechanical ventilation again. Due to severe acidosis as a consequence of acute respiratory failure, massive hemolysis occurred, with excess hemoglobinuria, resulting in dark brown urine. Plasmapheresis treatment was performed for two consecutive days to prevent kidney damage and initiate the remission of myasthenia gravis. After the second plasmapheresis, the complement protein 5 (C5) inhibitor eculizumab was administered in an induction dose of 600 mg intravenously. Two days after the first dose, the hemolysis completely stopped, and the haptoglobin level normalized. Before starting eculizumab therapy, C3, C4, and CH50 levels were below the lower limit, and one week after the first dose of the drug, complement levels were in the normal range, and the total complement activity was undetectable as a sign of the effective inhibition of a membrane attack complex. The events are summarized in Figure 1. No further evidence of hemolysis was observed, and the patient became transfusion-independent rapidly, but myasthenia-associated symptoms did not improve. Five days after the first dose, the second dose of eculizumab (300 mg) was administered. Flow cytometry monitoring revealed decreased clone size. The PNH clone was detectable in 20% of the erythrocyte population, possibly due to previous transfusions. Although mechanical ventilation could be temporarily discontinued, muscle strength decreased again, and expectoration difficulties developed, necessitating reintubation due to mucus plugs and incipient left lower lobe pneumonia.

As a consequence of this and the immunosuppression and complement inhibition, left lower lobe pneumonia progressed, and fulminant sepsis developed. Despite complete intensive care treatment, the patient died 10 days after the initiation of eculizumab therapy. An autopsy confirmed pneumonia and sepsis as the causes of death. No underlying malignancy was detected.

## 3. Discussion

The patient reported in this paper is unique as no other case had been reported where the co-existence of acetylcholine receptor antibody-positive myasthenia and paroxysmal nocturnal hemoglobinuria complicated the patient’s clinical case. In this case, the pathophysiology mechanism of both diseases involved the complement system. The initial activation step is different in the two diseases as, in myasthenia, the autoantibodies initiate the complement activation, whereas, in PNH, the membrane defect makes the cells prone to complement-mediated lysis. Thus, the available complement-blocking therapy may benefit both diseases [15]. The complement-blocking monoclonal antibody eculizumab had also been approved in MG and PNH, showing marked responses. Having suffered from myasthenia for 20 years, our patient developed hemolysis and PNH with type III cells diagnosed. As myasthenia was acetylcholine receptor antibody-positive and was not responding adequately to treatment, eculizumab was a logical treatment choice. The recent severe infection and the application of mechanical ventilation were not optimal circumstances for complement-blocking therapy. However, after the patient improved and was also diagnosed with PNH, both diseases required treatment [3,13,16,17]. The only logical and effective treatment choice was complement-blocking therapy. During the process of eculizumab individual permit application, she developed another myasthenia crisis, with severe hypoxia and acidosis that resulted in extreme hemolysis and massive hemoglobinuria. After two plasmapheresis treatments and transfusions, the administration of eculizumab promptly stopped the hemolytic process, and myasthenia started to improve. This is a reported effect of this therapy, but usually, slower responses are observed. The rapid response of hemolysis is a unique report from our case. It is known that MG requires several doses of eculizumab to be stabilized, and bulbar-type myasthenia responds less to C5 inhibition than the generalized type. She could only receive two weekly doses of eculizumab as she developed another pulmonary infection, necessitating ventilatory support again, and she died. The difference in the therapeutic response time is due to the slightly different mechanisms of the diseases. As PNH is only mediated by complement activation, blocking the complement system can have prompt effects, whereas, in myasthenia, the autoantibodies activate the complement, and blocking the complement has no effect on the antibody’s presence. Our case demonstrates that eculizumab has a powerful prompt effect on hemolysis control in PNH. Unfortunately, we could not keep the patient alive for the myasthenia to improve. The unique association of PNH with myasthenia may be accidental, but a common pathophysiology mechanism is also possible.

## Data Availability

The raw data supporting the conclusions of this article will be made available by the authors on request.
